# Supplementary material for: Ultrafast isomerization in acetylene dication after carbon K-shell ionization
Source: Nat Commun. 2017 Sep 6;8:453. doi: 10.1038/s41467-017-00426-6 (PMC5587545; doi:10.1038/s41467-017-00426-6)
Supplement: Supplementary file 1 — Supplementary Information [file 41467_2017_426_MOESM1_ESM.pdf]

Type of file: pdf

Title of file for HTML: Supplementary Information

Description: Supplementary Note, Supplementary Figures, and Supplementary References

------------------

Type of file: pdf

Title of file for HTML: Peer Review File

**Supplementary Note 1. The electronic structure and dynamics of the core-ionized acetylene cation**

We model the electronic structure of the core ionized acetylene cation $[DCCD]^+$ with a ΔSCF method[1] using the PBE0 exchange-correlation functional and the 6-31G* basis set. The core-hole is assumed to be localized on one of the carbon atoms due to coupling with non-totally symmetric modes.[2] The electronic potential energies and gradients are computed using GAMESS. In short, a closed-shell Kohn-Sham equation is solved to provide the initial orbitals for ΔSCF orbital optimization. The core shell orbitals are then localized on each carbon atom, respectively. A self-consistent field (SCF) procedure then follows with a singly occupied C1s core orbital. The SCF procedure is carried out

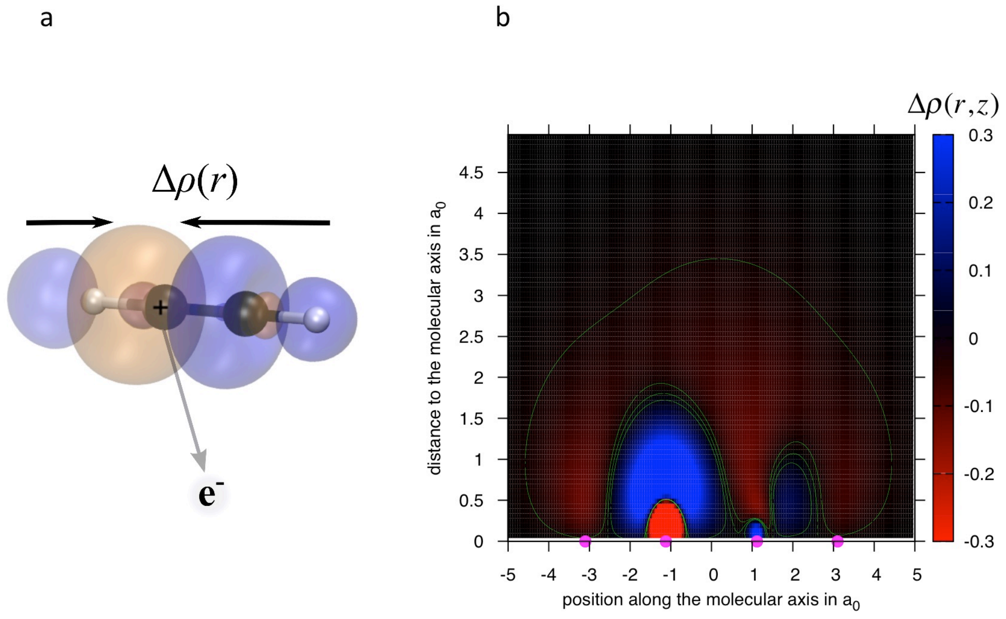


**Supplementary Figure 1. Electronic screening after core ionization.** (a) Sketch of the screening dynamics. The C1s core hole, as a positive charge, attracts valence electrons in the electronic relaxation after core photoionization. The orange/blue surfaces represent positive/negative increments in the total electron density $\Delta\rho(\mathbf{r})$ between the fully relaxed electronic structure, $\rho_{\mathrm{screen}}$, and that with a pure C1s core hole in Koopmans' sense, $\rho_0$. (b) 2D illustration of $\Delta\rho(r,z)$ in cylindrical coordinates, the positions of carbon and deuterium atoms are labeled with dots on the x-axis, and $\Delta\rho(r,z) = 2\pi r\left(\rho_{\mathrm{screen}}(r,z) - \rho_0(r,z)\right)$.

with the maximum overlap method to preserve the character of the core hole singly-occupied orbital. In the maximum overlap method, the occupied orbitals in each new SCF cycle are reordered and phased to maximize the overlap with the occupied orbitals of previous SCF cycle. The overlap matrix of MOs from the *k*-th and *k*+1-th SCF cycles is defined as

$$O_{ij}^{k,k+1} = \left\langle \varphi_i^k \middle| \varphi_j^{k+1} \right\rangle = \sum_{pq} C_{ip}^k S_{pq} C_{jq}^{k+1} \tag{1}$$

The ordering and phase of the MOs is adjusted to maximize the coincidence of the MOs between the *k*-th and *k*+1-th SCF cycles. The phase can be adjusted for any of the MOs (multiplying by ±1), but reordering is only allowed for orbitals with the same occupation number.

The dynamic screening effect of valence electrons is shown in Supplementary Figure 1, depicting the difference of the electron density between the core-ionized cation (with one electron removed from a localized C1s orbital) and the neutral molecule. We can estimate the time scale of the corresponding shake-up core hole relaxation after core-ionization and build-up of dynamical correlation from the C1s electron binding energy using the time-energy uncertainty principle as $t = 2\pi / E_{1s} \approx 25$ as . As shown in Supplementary Figure 1a, the net effect of core hole relaxation is to increase the valence bonding electron density in the region of the C-C bond, an increase which is due to attraction by the positively charged core hole. The C-C bond is thus strengthened, and the acetylene cation $[HCCH]^+$ has a shorter equilibrium C-C bond length than the neutral acetylene molecule, as shown in Supplementary Figure 2e. It is also a direct consequence of the valence electron screening effect that the angular potential along $\theta_{CCD}$ coordinate is hardened at shorter C-C bond length $R_{CC}$, because bonding along the C-C axis is

strengthened (see Supplementary Figure 2b). However, the angular potential for fixed C-C bond length shows a slight softening (see Supplementary Figures 2c and 2d).

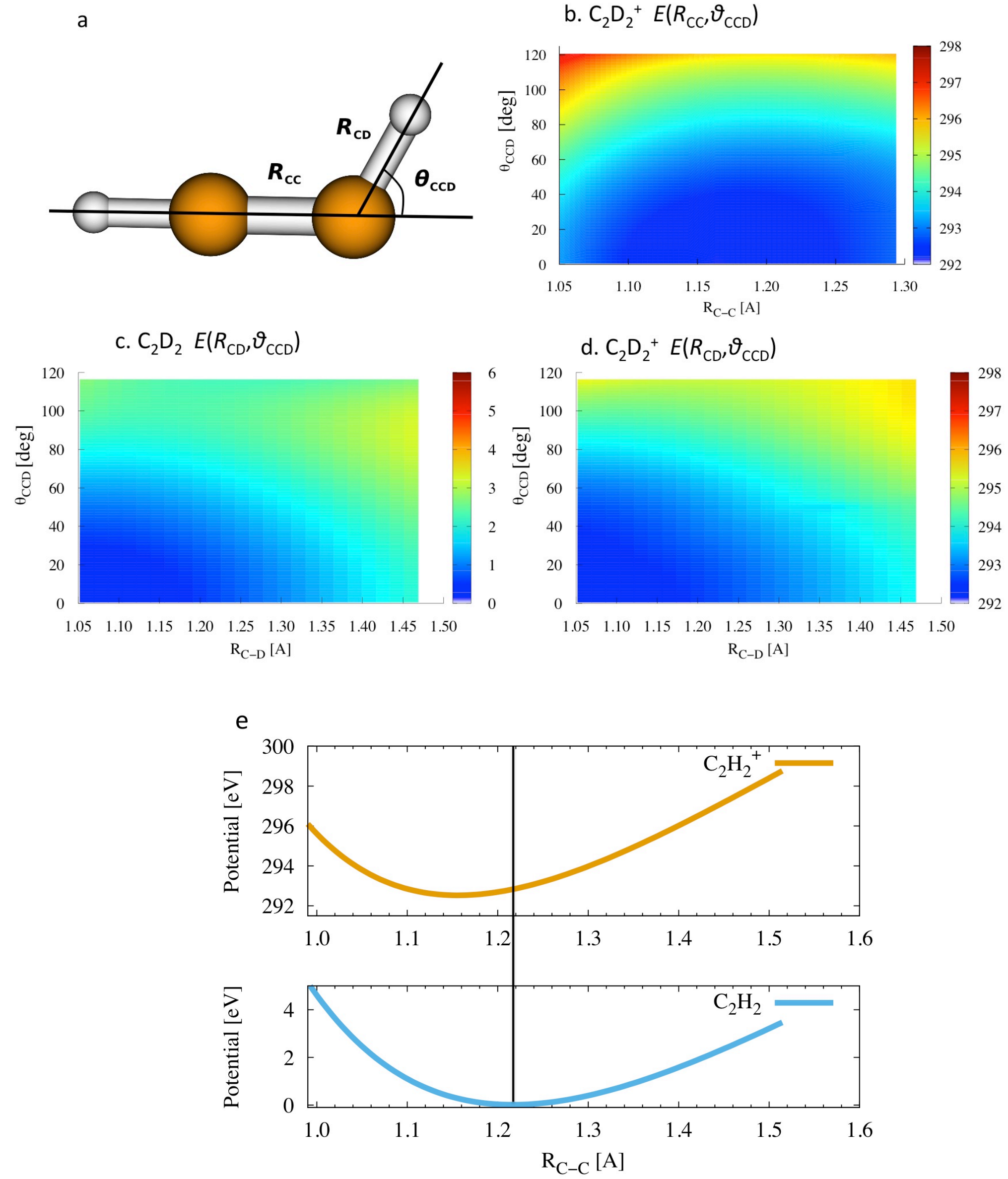


**Supplementary Figure 2. Potential energy surface of the $^2\Sigma$ C1s core ionized acetylene cation.** (a) Illustration of the coordinates $R_{CC}$, $R_{CD}$ and $\theta_{CCD}$. (b) 2D potential $E(R_{CC}, \theta_{CCD})$ of core ionized acetylene cation with $R_{CD}$=1.06Å. (c) 2D potential $E(R_{CD}, \theta_{CCD})$ of neutral acetylene molecule on $^1\Sigma_g$ state with $R_{CC}$=1.22Å. (d) 2D potential $E(R_{CD}, \theta_{CCD})$ of core ionized acetylene cation on $^2\Sigma$ state with $R_{CC}$=1.22Å. (e) 1D potential $E(R_{CC})$ of core ionized acetylene cation on $^2\Sigma$ state with $R_{CD}$=1.06Å and $D_{\infty h}$ symmetry.

**Supplementary Note 2. Auger decay and generation of acetylene dication**

The Auger decay rates are computed along the trajectories from a single Slater determinant wave function built with Kohn-Sham orbitals. The amplitude of Auger decay with an initial core hole in orbital $\phi_c$ to a final electronic configuration with double valence hole in orbitals $\phi_a$, $\phi_b$ and an Auger electron wave function $\phi_k$ with momentum $k$ can be determined as[3]

$$A\left(\mathrm{i}[c]\rightarrow \mathrm{f}[ab][k]\right)\sim\left(\langle ab|ck\rangle\delta_{\sigma_a\sigma_c}\delta_{\sigma_b\sigma_k}-\langle ba|ck\rangle\delta_{\sigma_b\sigma_c}\delta_{\sigma_a\sigma_k}\right)\langle\Psi_f^N|\hat{c}_a^\dagger\hat{c}_b^\dagger\hat{c}_c|\Psi_i^{N-1}\rangle|k\rangle, \quad \textbf{(2)}$$

where $\langle ab|ck\rangle$ is defined as

$$\langle ab|ck\rangle=\iint dr_1 dr_2\ \phi_a(r_1)\phi_b(r_2)\frac{1}{|r_1-r_2|}\phi_c(r_1)\phi_k(r_2). \quad (3)$$

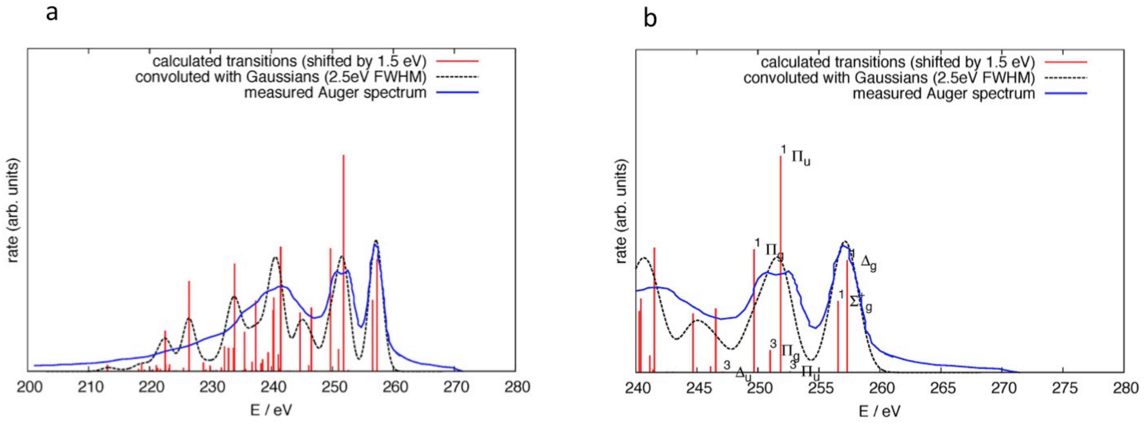


**Supplementary Figure 3. KLL Auger spectrum of acetylene molecule.** (a) Spectrum over wide energy range, simulated using single-center expansion method at equilibrium geometry subject to primary carbon K-shell photoionization.[4] The broadening width is taken to be 2.5 eV in order to match the experimental[5] data. The spectrum is dominated by the Auger decay into singlet dicationic states, with an Auger lifetime of ~8 fs. (b) Same spectrum as in (a), but zoomed in to a narrower energy range and with assignments for lowest energy peaks.

Based on the fact that the core hole orbital $\phi_c$ is strongly localized on the atom subject to primary photoionization, the molecular continuum wave function $\phi_k$ in the two-electron integral can be approximated with the atomic continuum wave function $\phi_{\varepsilon lm}$ with ε the energy of the atomic Auger transition and $l$,$m$ the angular quantum numbers. Employing

this approximation, the two electron integrals are approximated using the LCAO expansion only on the atom on which the core hole is localized[6]

$$\langle ab|ck\rangle = \sum_{\mu\nu\lambda,\text{ on atom}} \sum_{lm} C_{\mu a} C_{\nu b} C_{\lambda c} \langle \mu\nu|\lambda;\varepsilon lm\rangle,\ \varepsilon = \varepsilon_\mu + \varepsilon_\nu - \varepsilon_\lambda = \frac{k^2}{2}. \quad (4)$$

Assuming frozen molecular orbitals and neglecting any configurational mixing, the Auger rates are calculated for the singlet (S) and triplet (T) final states as

$$\begin{aligned}\Gamma_S\left(\mathrm{i}[c]\rightarrow \mathrm{f}[ab][\varepsilon]\right) &= 2\pi \sum_{lm} N_{ab}\left[\frac{1}{2}\left|\langle ab|c;\varepsilon lm\rangle + \langle ba|c;\varepsilon lm\rangle\right|^2\right],\\ \Gamma_T\left(\mathrm{i}[c]\rightarrow \mathrm{f}[ab][\varepsilon]\right) &= 2\pi \sum_{lm} N_{ab}\left[\frac{3}{2}\left|\langle ab|c;\varepsilon lm\rangle - \langle ba|c;\varepsilon lm\rangle\right|^2\right],\end{aligned} \quad (5)$$

where $N_{ab} = 1,\ a \neq b$ and $N_{ab} = 1/2,\ a = b$. For the evaluation of atomic two electron integrals we employ the XATOM code.[7] The use of a minimal basis allows a direct identification of basis functions with atomic orbitals.

A simulated Auger spectrum at the Franck-Condon geometry is presented in Supplementary Figure 3. The spectrum is computed with the single center expansion method,[4] where the double hole states after Auger decay are described by wave functions determined from multi-reference configuration interaction (MRCI).

From the simulated Auger spectrum in Supplementary Figure 3, we could determine the FWHM of the experimental spectra to be ~2.5eV[5]. In the coincidence experiment,[8] the Auger electrons that correlate with the putative isomerization channel have a distribution centered at 255.5 eV.[8] These Auger electrons could have three sources, from the low energy wing of the $1\pi_u^{-2}$ states ($^1\Delta_g$, $^1\Sigma_g$) centered at 257.1±0.2eV, from the high energy wing of the $1\pi_u^{-1}3\sigma_g^{-1}$ states ($^1\Pi_u$) centered at 252.6±0.4eV, and from the 3h1p satellite state ($^1\Sigma_u$) with Auger energy of 256.8 eV that is accessed via photoionization and shake-up process. The pathways on the $^1\Pi_u$ and $^1\Sigma_u$ states were

assumed to support the purported ultrafast isomerization on the sub-100 fs time scale,[9] and the pathways on the $^{1}\Delta_g$ and $^{1}\Sigma_g$ states are open for isomerization on a longer time scale that corresponds to the dynamics of potential barrier crossing. These channels all together contribute to the measured vinylidene-like signals in Refs. 8,10.

For dynamical Auger spectra, we adopt the single determinant ΔSCF method for computational efficiency. The Auger spectra show dominant decay channels into singlet states, and a cumulative Auger lifetime of ~8 fs. Within the Auger lifetime, it is sufficient for the geometrical relaxation to take place on the cationic $^{2}\Sigma$ state, that the acetylene cation evolves towards a linear structure with shorter C-C bond length. The Auger rates computed at each step along the trajectories are used to construct initial conditions of dynamics for the dication through solution of the corresponding kinetic rate equation.

## Supplementary Note 3. Dynamics on the dicationic states and Coulomb explosion imaging

### A. Dicationic potentials

The electronic structure of acetylene dication during the ab initio multiple spawning molecular dynamics is computed using the dynamically weighted[11] SA8-CASSCF(8,8)/6-31G* method, which is quantitatively consistent with MS8-CASPT2(8,10)/6-31G*. In Supplementary Figure 4, we present the diabatic potential of the dication along the trans-bending coordinate $\theta_{CCD}$. The potential curve of the $^{1}\Sigma_u$ state cuts through those of the $^{1}\Pi_g$ and $^{1}\Pi_u$ states as the C-C bond stretches (Figure 1 in the main text). The $^{1}\Sigma_u$, $^{1}\Pi_g$ and $^{1}\Pi_u$ states together form a low-barrier pathway for isomerization in the corresponding adiabatic states.

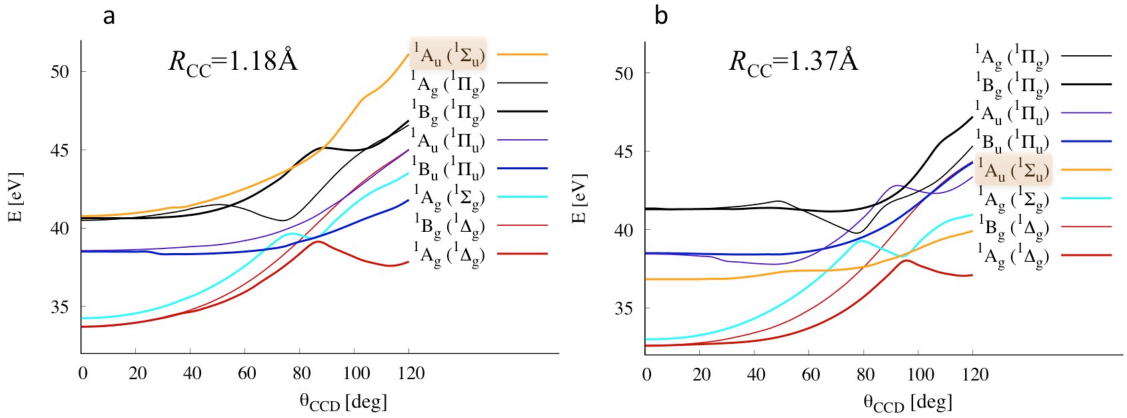


**Supplementary Figure 4. 1D diabatic potentials $E(\theta_{CCD})$ of acetylene dication.** Potential energy surfaces are calculated at the SA8-CASSCF(8,8)/6-31G* level, with $R_{CD}$=1.12Å and $R_{CC}$=1.18Å, 1.37Å, i.e. the C-C bond lengths of the Franck-Condon geometry and the equilibrium C-C bond length of the dicationic ground state. The electronic characters are labeled by the representations of $C_{2h}$ symmetry and that correlated with linear geometry (in parenthesis, $\theta_{CCD}=0^\circ$, $D_{\infty h}$ symmetry). At the Franck-Condon geometry, the $^1\Delta_g$ and $^1\Sigma_g$ states that correlate with adiabatic states $S_0$-$S_2$ are of $1\pi_u^{-2}$ character, the $^1\Pi_u$ and $^1\Pi_g$ states that correlate with adiabatic states $S_3$-$S_4$ are of electronic character $1\pi_u^{-1}3\sigma_g^{-1}$ and $1\pi_u^{-1}2\sigma_u^{-1}$. The $^1\Sigma_u$ state is a 3-hole-1-particle (3h1p) satellite state with electronic character $1\pi_u^{-2}+(\pi_u \rightarrow \pi_g^*)$, it correlates initially with $S_7$ at Franck-Condon geometry (a), and becomes $S_3$ as the C-C bond stretches (b). It is noticeable that the potential of $^1\Sigma_u$ state flattens along the $\theta_{CCD}$ coordinate as the C-C bond relaxes. The zero of energy is set as the ground state energy of acetylene at its equilibrium geometry.

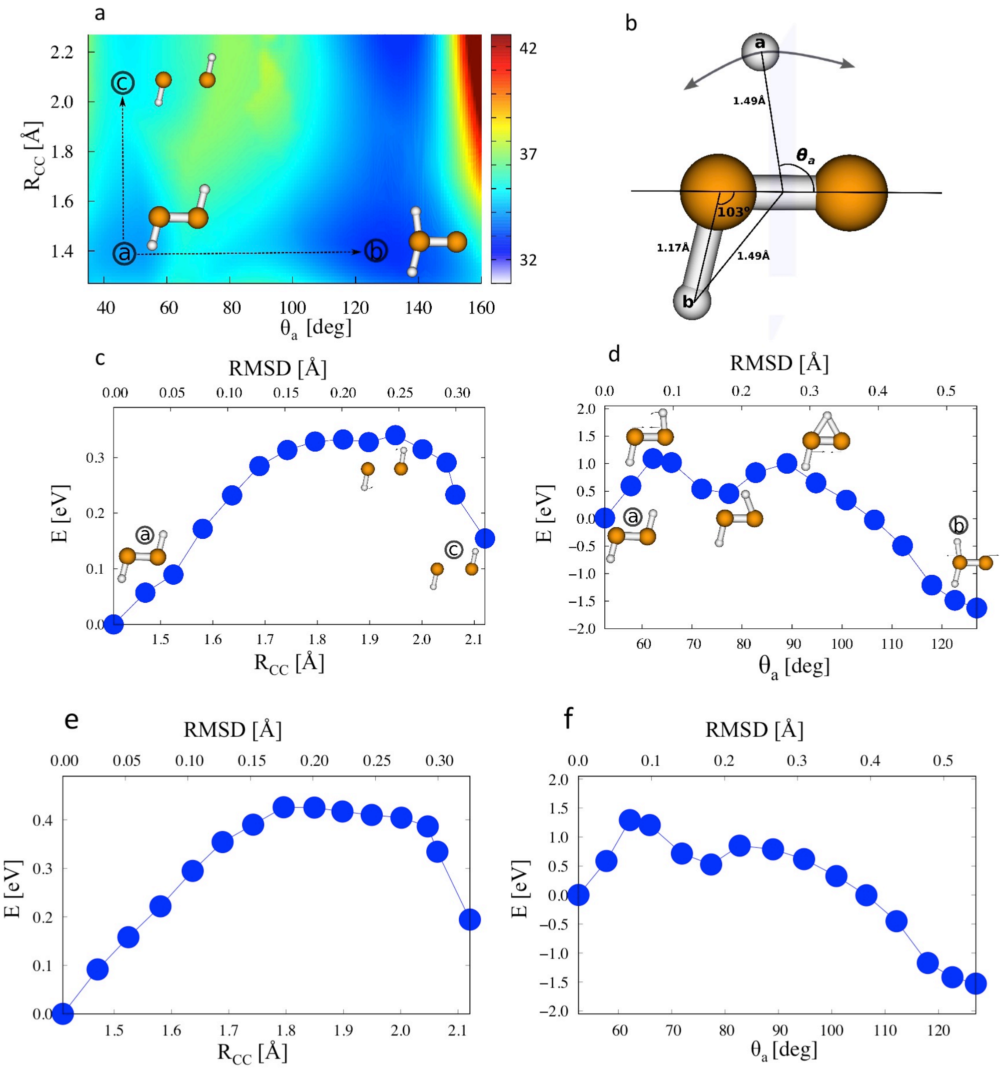


**Supplementary Figure 5. Isomerization pathway on the $1\pi_u^{-2}$ state $S_2$.** Geometry changes on $S_2$ are crucial to complete the isomerization when the dication decays from $1\pi_u^{-1}3\sigma_g^{-1}$ states. (a) 2D potential $E(R_{CC},\theta)$ from scan with rigid geometry on the remaining degrees of freedom. (b) Illustration of degrees of freedom for rigid potential scan in (a), deuteron $D_b$ is fixed at the position from optimized geometry of $[CD_2C]^{2+}$. (c) Potential along C-C fragmentation pathway with relaxed geometry. (d) Potential along isomerization pathway with relaxed geometry. (e), (f) Potential along C-C fragmentation (e) and isomerization (f) pathways with relaxed geometry at the MS8-CASPT2(8,10) level.

In Supplementary Figure 5, we present the potential along C-C fragmentation and isomerization pathways on the $1\pi_u^{-2}$ state $S_2$, which is crucial to complete isomerization process by providing a bounded C-C potential for the dication. We optimize the geometry on the excited dicationic states for the isomerized and fragmented dication, and calculate

the potential along the isomerization and fragmentation coordinates. Supplementary Figure 5c shows a bound potential for the C-C stretch coordinate with a barrier of ~0.3 eV, and in Supplementary Figure 5d, we show that isomerization requires formation of a triangular C-D-C bridge as the deuteron migrates halfway to the middle of the two carbon atoms, with the two carbon atoms contracting towards each other. This however imposes another disadvantageous factor to the isomerization channel, because the C-C stretch

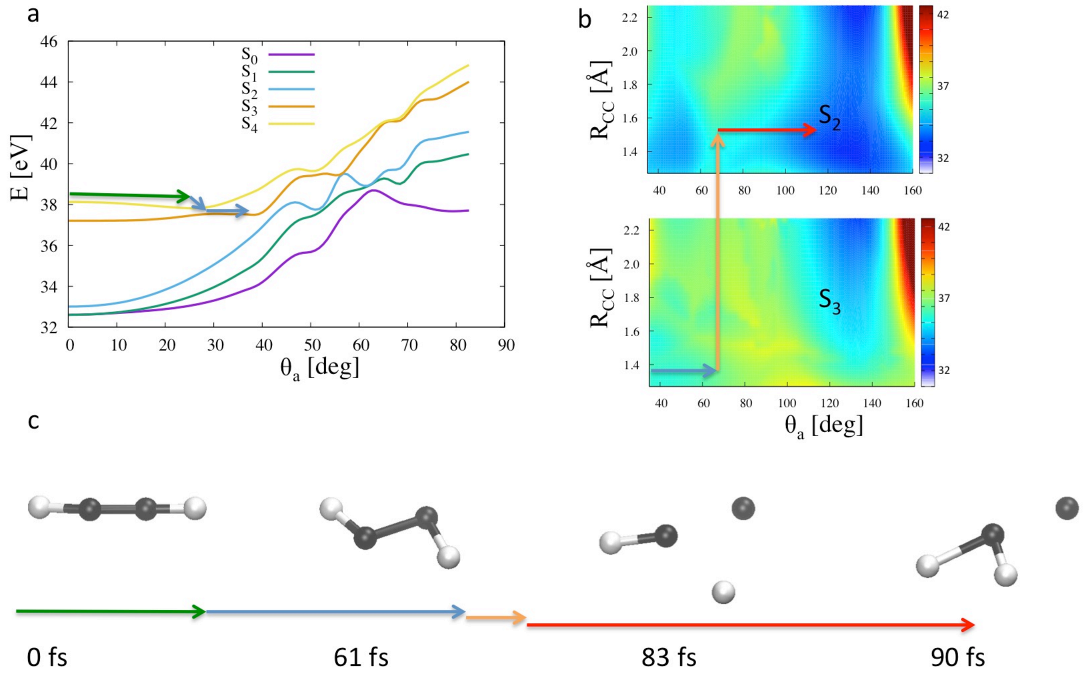


**Supplementary Figure 6. Schematic of a possible sub-100 fs isomerization pathway.** (a) trans-bending potential plotted with $\theta_a$ coordinate and assuming $\theta_b=\pi+\theta_a$, where the angle $\theta_a$ is described in Supplementary Figure 5b. The trajectory (c) starts from the high lying $1\pi_u^{-1}3\sigma_g^{-1}$ states $S_4$ (a) that allows the deuteron to move to a large angle before 60 fs, the non-Born-Oppenheimer transition downwards to $S_3$ and $S_2$ offers a bounded potential for the C-C bond (b), the dication then isomerizes on the low lying $1\pi_u^{-2}$ states at ca. 90 fs. In (a) the potentials are calculated with $R_{CD}=1.12$Å, $R_{CC}=1.37$Å and trans-bending angle $\theta_{CCD}$, in (b) they are calculated with $R_{CM\text{-}D}=1.49$Å, $R_{CC}=1.37$Å, and deuteron migration angle $\theta_a$, $R_{CM\text{-}D}$ is the distance of the migrating hydrogen to the center of mass (CM) of the two carbon atoms, and we define $\theta_a=\theta_{C\text{-}CM\text{-}D}$. The arrow in (a) illustrates the direction of the isomerization, and the arrow in (b) shows the pathway of electronic decay through the conical intersection from $S_3$ to $S_2$ that occur at the end of the isomerization process. Note that although this pathway is possible, the simulations predict it to be highly improbable.

motion is kinematically enhanced due to kinetic energy release from geometric relaxation after Auger decay. Benchmark calculations at the MS8-CASPT2(8,10)/6-31G* level in Supplementary Figures 5e and 5f show the reliability of the *ab initio* potentials obtained on the SA8-CASSCF(8,8)/6-31G* level for the AIMS dynamics.

B. Ab initio multiple-spawning (AIMS) molecular dynamics simulation

The coupled electron-nuclear dynamics of acetylene dication is simulated with the ab initio multiple spawning (AIMS) method,[12] which solves the electronic and nuclear Schrödinger equations simultaneously using a travelling Gaussian wavepacket basis for the nuclear wave function and electronic structure methods at various levels (dynamically-weighted SA-CASSCF in this work). The total electronic nuclear wave function is represented by a time-dependent basis set of atom-centered Gaussians for the nuclear degrees of freedom as

$$\boldsymbol{\Psi}(R,r,t)=\sum_{I}\sum_{i}^{N_I(t)} c_i^I(t)\chi_i^I(R;\bar{R}_i^I,\bar{P}_i^I,\gamma_i^I)\phi_I(r;R), \tag{6}$$

where I labels electronic states, $N_I(t)$ is the number of nuclear basis functions associated with the Ith electronic state at time t, and r/R label electronic/nuclear coordinates respectively. The nuclear basis increases during the simulation when nonadiabatic coupling increases and electronic transitions are possible, which is dubbed *spawning*, and the nuclear Schrödinger equation is solved in this basis to determine the time evolution of electronic population. The nuclear basis functions $\chi_i^I(R;\bar{R}_i^I,\bar{P}_i^I,\gamma_i^I)$ are parameterized by the positions/momenta in phase space and a phase factor $\gamma_i^I$. The electronic basis functions $\phi_I(r;R)$ are solved using the GPU-based electronic structure program TeraChem[13,14] in the adiabatic representation at the SA8-CASSCF(8,8)/6-31G* level to provide electronic

potential energies, gradients and non-Born-Oppenheimer couplings at each step. The complex coefficients $c_i^I(t)$ evolve following the time-dependent Schrödinger equation (TDSE) in the time-dependent basis, i.e.:

$$\sum_{kK} S_{jk}^{JK} \dot{c}_k^K = -i \sum_{kK} (H_{jk}^{JK} - i\dot{S}_{jk}^{JK}) c_k^K \tag{7}$$

The overlap matrix, its time derivative and the Hamiltonian matrix elements are defined as:

$$\begin{aligned} S_{jk}^{JK} &= \left\langle \chi_j^J \phi_J \middle| \chi_k^K \phi_K \right\rangle \delta_{JK}, \\ \dot{S}_{jk}^{JK} &= \left\langle \chi_j^J \phi_J \middle| \frac{\partial \chi_k^K}{\partial t} \phi_K \right\rangle \delta_{JK}, \\ H_{jk}^{JK} &= \left\langle \chi_j^J \phi_J \middle| \hat{H} \middle| \chi_k^K \phi_K \right\rangle. \end{aligned} \tag{8}$$

The observables O(t), such as positions and momenta of atoms, for the Ith electronic state are calculated as

$$\left\langle O(t) \right\rangle_I = \frac{\sum_k^{N_I(t)} c_k^{I*} c_k^I \left\langle \chi_k^I \phi_k \middle| \hat{O} \middle| \chi_k^I \phi_k \right\rangle}{\sum_k^{N_I(t)} c_k^{I*} S_{kk}^{II} c_k^I} \tag{9}$$

The observables averaged over all electronic states can be obtained from incoherent summation (for operators which are diagonal in the electronic state index) over the electronic states as

$$\left\langle O(t) \right\rangle = \sum_I \rho_I(t) \left\langle O(t) \right\rangle_I \tag{10}$$

where $\rho_I(t)$ is the population of *I*th electronic state at time *t*.

C. Dynamics of dication

The recoil momentum of the outgoing Auger electron with energy of ca. 255 eV and the angular momentum of the trans-bending motion can set the $[C_2D_2]^{2+}$ dication into rotation, and this rotation has been used as a clock to estimate the time of isomerization.[10]

In Supplementary Figure 7, we show the rotational motion of the C-C axis from the molecular dynamics simulation. The existence of rotation is consistent with the previous observation, however the dications only rotate for ~0.15 rad in 100 fs, slower than expected in Ref. 10, and the dispersion of the $\theta_{CC}$ angle distribution also shows a deceleration. Because the C-C bond stretches from 1.17Å to 1.37Å, the angular velocity $\omega$ decelerates due to conservation of angular momentum. Additionally, the recoil momentum from an Auger electron of 255 eV adds at most ~0.012 eV of kinetic energy to the bending motion of the dication when it acts normal to the C-C axis. The recoil motion has very limited influence on isomerization in comparison with the total kinetic energy of 0.95 eV, and it is especially hard to compete with the 0.43 eV of kinetic energy in the C-C stretching motion (which favors the symmetric fragmentation channel). For the lowest three singlet states where the σ-orbital remains doubly occupied, the unidirectional σ-bond along the C-C axis should weaken the ability of recoil momentum to cause isomerization. Furthermore, the Auger electron from delocalized valence orbitals will impose recoil momentum to all atoms, further diminishing its ability to promote isomerization. Thus we suggest that the isomerization due to barrier crossing on the lower $1\pi_u^{-2}$ dicationic state takes place at much longer time scales, which is consistent with the conclusion from an energetic perspective,[15] and also with the observed dynamics in AIMS simulations.

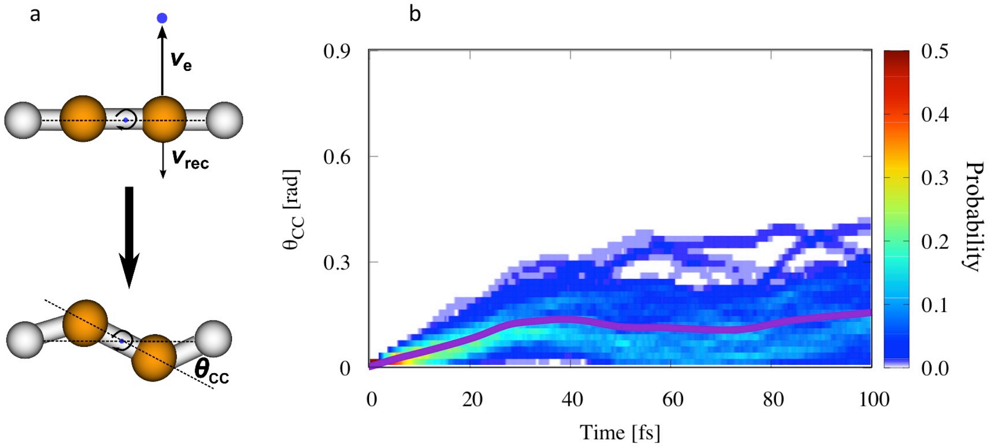


**Supplementary Figure 7. Rotational motion of C-C axis after Auger decay.** (a) Sketch of rotation of C-C axis with initial velocity $v_{rec}$ due to recoil momentum of Auger electron with velocity $v_e$ on the lever, and possible supporting effect the C-C rotational motion to isomerization. (b) Rotation of C-C axis in the lab system due to recoil momentum of Auger electron, calculated from molecular dynamics simulation, where the rotation angle is defined as $\theta_{CC}(t) = \left| \cos^{-1}\left( \frac{\vec{R}_{CC}(t) \cdot \vec{R}_{CC}(0)}{|R_{CC}(t)||R_{CC}(0)|} \right) \right|$. The solid curve corresponds to $\langle \theta_{CC} \rangle (t)$.

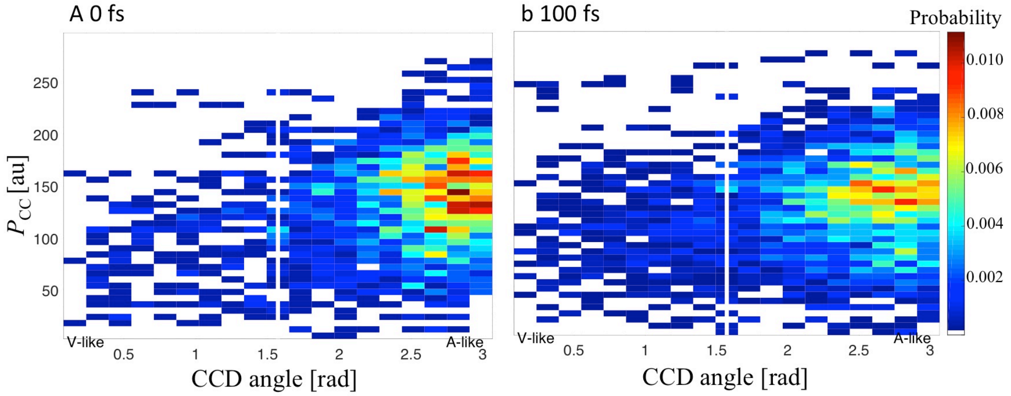


**Supplementary Figure 8. Four-particle coincidence Coulomb momentum mapping.** Experimental momentum difference data for $C^+$ ions in four-particle coincidence Coulomb momentum mapping. (a) coincidence counts at 0fs. (b) coincidence counts at 100fs.

In Figure 3 of the main text, we present geometric configurations of the dication that can be accessed by the ensemble of nuclear trajectories from the ab initio multiple spawning molecular dynamics simulation, where no significant isomerization can be detected. The most compelling argument against isomerization is that direct simulation does not produce significant isomerization while the resulting data is simultaneously in

agreement with the experimental observations. However, it is also of interest to show that this result can be rationalized with a simplified analysis, which we do here. We calculated the kinetic energy distribution in the C-C stretching separately and the remaining 5 degrees of freedom, because the C-C stretching mode is the dominantly excited mode in the dication formation. Because both C-C stretch and trans-bending motion are quasi barrier free on the $1\pi_u^{-1}3\sigma_g^{-1}$ states, we can assume all the occupied states of these two degrees of freedom correspond to open channels. We can estimate the branching ratio between C-C fragmentation and isomerization simply from probability of open channels $P_i = g\frac{(2\pi m_i kT)^{3/2}V}{h^3}e^{-\frac{E_i}{kT}}$, where $m_i$ and $g$ are the reduced mass and degeneracy of the corresponding mode, respectively, and $V$ is the quantization volume. Assuming ambient temperature $T$=300 K, the kinetic energy of C-C stretch is obtained from the AIMS trajectories for the 100 fs dynamics as $\langle E_{\mathrm{KER,CC}}\rangle = \frac{1}{T}\int dt\, E_{\mathrm{KER,CC}}(t) \sim 0.43\ \mathrm{eV}$, while the total kinetic energy is given as $\langle E_{\mathrm{KER}}\rangle = \frac{1}{T}\int dt\, E_{\mathrm{KER}}(t) \sim 0.95\ \mathrm{eV}$. The reduced masses for C-C stretch and trans-bending motion are taken as 7 amu and 2.592 amu from spectroscopic data.[16] For simplicity, we assume all recoil energy of 0.012 eV is pumped into the trans-bending degrees of freedom (DOF), which will overestimate the likelihood of trans-bending. The rest of the kinetic energy $\langle E_{\mathrm{KER}}\rangle - \langle E_{\mathrm{KER,CC}}\rangle$ is equally distributed among the five DOFs. Of course, this is only an estimate since IVR is expected to complete on the picosecond timescale. We have then $E_{\mathrm{KER,TB}} \sim \frac{2}{5}\times(0.52-0.012)+0.012=0.215\ \mathrm{eV}$ for the trans-bending mode. The ratio between the isomerization and C-C fragmentation channels is then estimated to be $1.1\times10^{-4}$.

Figure 3d presents an angular distribution of the deuterons, which is plotted so that the eye can more easily compare it to the momentum distribution. First, the angular distribution is drawn on an annulus that is comparable the momentum distribution seen in the experiment. Then, the distribution is given a Gaussian width, again to mimic the momentum distribution seen in the experiment.

In principle, intersystem crossing might be relevant, and the isomerization might occur on a triplet state. In order to address this possibility, we have determined the largest spin-orbit coupling matrix element for acetylene dication at the SA8-CASSCF(8,8)/6-31G* level to be 3.21 meV between the $\left|{}^{1}\Delta_g; \Lambda = \pm 1, M_S = 0, M_J = \pm 1\right\rangle$ and $\left|{}^{3}\Pi; \Lambda = \pm 1, M_S = 0, M_J = \pm 1\right\rangle$ states, which corresponds to a time scale of 1.29ps. This makes intersystem crossing from the singlet to triplet states in acetylene dication dynamics quite improbable on the sub-100fs time scale. Here $\Lambda$, $M_S$ and $M_J$ are the projections of the orbital, spin and total angular momenta, respectively.

A further possibility to consider is the impact of tunneling on these results. We are confident that tunneling isomerization can be safely excluded from our scenario. Subject to a potential barrier of ~2eV and heavy deuteron isotope, the tunneling isomerization should take place on microsecond (μs) time scale,[17] which is substantially longer than the sub-100fs time scale.

D. Coulomb momentum mapping

The Coulomb momentum mapping by further core photoionization and Auger decay with an X-ray probe pulse of certain time delay produces a tetracation $[C_2D_2]^{4+}$. The Coulomb explosion of the tetracation is simulated by a classical Hamiltonian of four singly charged particles $C^+/C^+/D^+/D^+$,

$$
\begin{aligned}
&H = T + V_{\text{Coul}}(r_1, r_2, r_a, r_b) \\
&V_{\text{Coul}}(r_1, r_2, r_a, r_b) = \frac{1}{r_{1a}} + \frac{1}{r_{1b}} + \frac{1}{r_{ab}} + \frac{1}{r_{12}} + \frac{1}{r_{2a}} + \frac{1}{r_{2b}},
\end{aligned}
\tag{11}
$$

because dynamics of the highly repulsive tetracation is not sensitive to its detailed electronic structure. In Eq. 12, $T$ is the kinetic energy, $r_1$, $r_2$, $r_a$, $r_b$ are the coordinates of the carbon and deuteron ions as shown in Supplementary Figure 9a, and $V_{\text{coul}}(r_1, r_2, r_a, r_b)$ is the Coulomb potential. An interatomic distance cutoff of 5 Å is set for atoms involved in the Coulomb explosion in order to guarantee the atoms subject to the same parent dication are charged by Auger decay induced by the X-ray probe pulse. Molecular dynamics of the tetracation $[C_2D_2]^{4+}$ is carried out for 5 ps. Final distances of ions are of $\sim 10^3$ Å, from which we can assume the Coulomb potential has been fully released. The deuteron momenta are projected on the C-C axis defined by the difference of momenta of the two carbon ions.

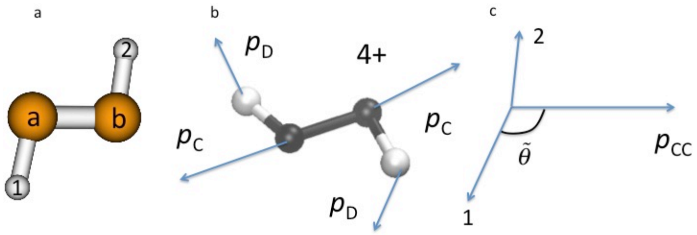


**Supplementary Figure 9. Schematic diagrams for Coulomb momentum mapping.** (a) Labeling of atoms in acetylene. (b) Labeling of momentum vectors. (c) Definition of angle which was used to separate acetylene-like and vinylidene-like signals (see Eq. 12).

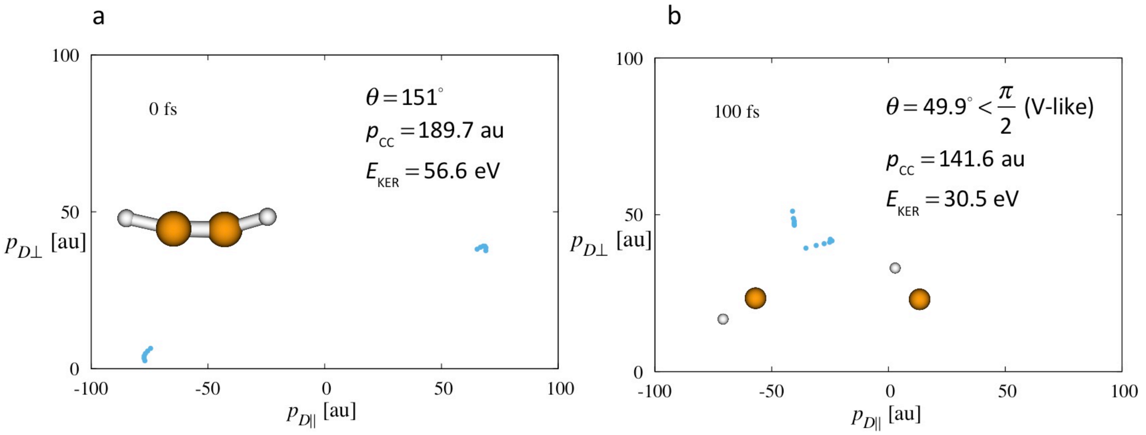


**Supplementary Figure 10. Simulated Coulomb momentum mapping.** Illustrative Coulomb momentum mapping data from a representative trajectory. X-ray probe pulse time delay of (a) t=0 fs and (b) t=100 fs. The trajectory gives V-like signal at time delay t=100 fs, even though it clearly belongs to the C-C fragmentation channel. The $CD^+$ ion fragment carries remnant angular momentum inherited from trans-bending motion before fragmentation. For each time delay t, we compute five deuteron momentum mappings from t-2 to t+2 fs.

To classify the four-particle coincidences signal, we define the angle $\tilde{\theta}$:[9]

$$\tilde{\theta} = \cos^{-1}\left( \frac{\mathrm{sgn}\left[\left(p_{C_a} - p_{C_b}\right)\cdot p_{D_2}\right]\left(\left(p_{C_a} - p_{C_b}\right)\cdot p_{D_1}\right)}{\left|p_{C_a} - p_{C_b}\right|\left|p_{D_1}\right|} \right) \tag{12}$$

We classify the $C^+/C^+/D^+/D^+$ four-particle-coincidence as vinylidene-like (V-like) for $\tilde{\theta} < \frac{\pi}{2}$, such that both deuteron momenta reside on the same side of the bisection plane that divides the C-C axis. The remaining coinicidences with $\tilde{\theta} > \frac{\pi}{2}$ are referred as acetylene-like (A-like) with deuteron momenta on opposite sides of the bisection plane. An important finding from our work is that V-like coincidence does not necessarily correspond to an isomerization event (at variance with previous assumptions).

Assuming a C-C bond length limit of dissociation to be 1.5 Å, and considering only the collinear Coulomb force between the two $C^+$ ions, an isomerized acetylene

dication should give $p_{C^+C^+}$ with a lower bound of ~181 au and V-like signal in the four-particle momentum map. Because the $C^+$ ions also feel the Coulomb force from the $D^+$ ions, the actual $p_{C^+C^+}$ should be higher than 181 au. In the non-isomerized trajectories of the molecular dynamics calculation that satisfy the lower bound, the lowest $\tilde{\theta}$ angle they can reach is 20°, with $p_{C^+C^+} = 199\,\text{au}$. In the V-like signals experimental data set, we attribute the four-particle coincidence signals with $\tilde{\theta} \leq 20^\circ$ and $p_{C^+C^+} \geq 199\,\text{au}$ to the isomerization channel, which is not covered by the MD trajectories.

In Supplementary Figure 8, we show the momentum difference $p_{C^+C^+}$ of $C^+$ ions measured in the time-resolved x-ray pump x-ray probe experiment at LCLS.[9] The ab initio multiple spawning molecular dynamics simulation sets a criterion of for V-like signals that do not come from isomerization channel and reinforces the fact that pathways of sub-100 fs isomerization mediated by the non-Born-Oppenheimer effect is a very rare channel. In fact, only one initial condition (out of 500) ever approached this channel (with ∠CCD>75°), and the transition probability was $\approx 1 \times 10^{-4}$. Thus, we estimate the probability of observing this channel to be less than $1 \times 10^{-6}$. Indeed, the only reason to mention this channel is that it seems the only feasible way that any sub-picosecond isomerization could occur in the experiment.

In Supplementary Figure 10, we show a representative trajectory of symmetric fragmentation channel with breakup of C-C bond, which gives V-like coincidence signal due to the remnant angular momentum of the $CD^+$ fragments. However, the C-C fragmented trajectory with V-like coincidence should have lower KER and $p_{CC}$, and can be distinguished from isomerization channel with V-like coincidence, because long C-C

distance simply implies low Coulomb potential release. Thus, we reconsider the measured Carbon momenta in the experiment with respect to the C-C momentum.

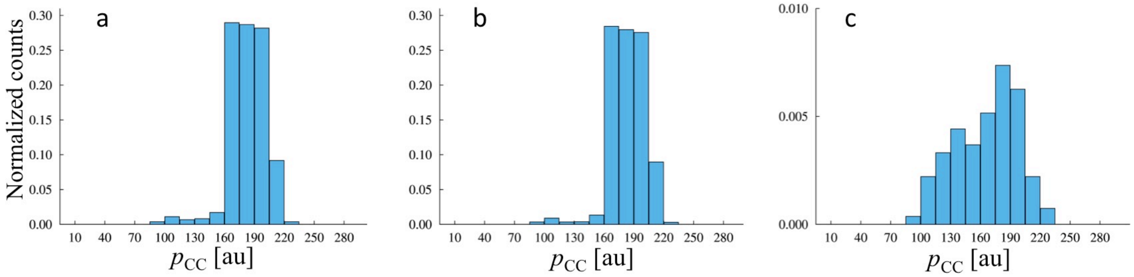


**Supplementary Figure 11. Simulated Coulomb momentum mapping.** Momentum difference of $C^+$ ions in the Coulomb momentum mapping at 100 fs from molecular dynamics calculations. (a) Total coinicidence counts. (b) Coincidence counts of A-like signals. (c) Coincidence counts of V-like signals.

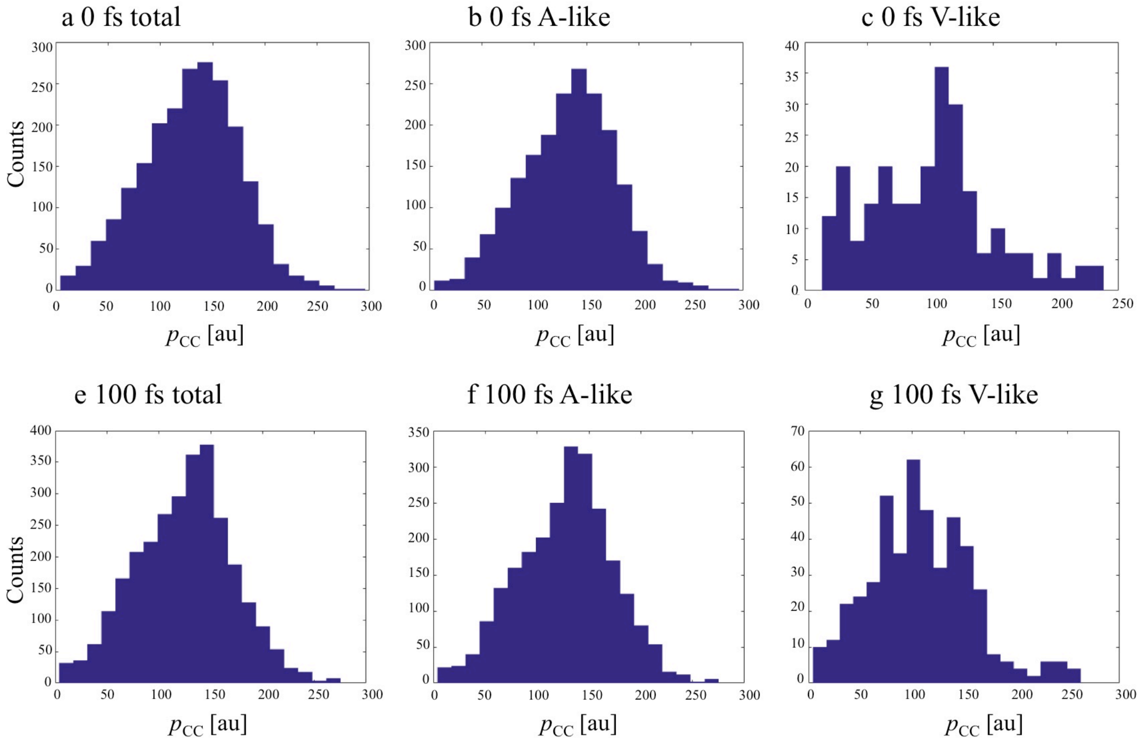


**Supplementary Figure 12. Experimental Coulomb momentum mapping.** Momentum difference of $C^+$ ions measured in Coulomb momentum mapping. Upper panels correspond to 0fs time delay and show (a) Total coincidence counts, (b) coincidence counts of A-like signals, and (c) coincidence counts of V-like signals. Lower panels correspond to 100 fs time delay and show (e) Total coinicidence counts, (f) coincidence counts of A-like signals, and (g) coincidence counts of V-like signals.

Supplementary Figures 11 and 12 show the momentum difference obtained in the experiment and from the MD simulations.

It is noticeable that there are coincidence counts in Supplementary Figure 12 with low momentum difference $p_{C^+C^+} \leq 70$ au that appear within 100 fs, these coincidence counts are not found in the molecular dynamics simulation of 120 fs, and they correspond to C-C distance larger than 30 Å, these coincidence count signals may come from the highly dissociative states above $S_7$, which we have not considered in the simulation and should also have negligible contribution to isomerization. Especially, the coincidence counts with very low momentum difference down to 10 au appear from 0 fs (Supplementary Figure 11): these signals correspond to C-C distances that could be larger than 1 μm, and are comparable to the size of the XFEL-sample interaction region of 50 $\mu m^2$.[9] They give a clear hint that despite filtering using the momentum conservation criterion:

$$\left| p_{D_{a,j}} + p_{D_{b,j}} + p_{C_{a,j}} + p_{C_{b,j}} \right| = p_j < \delta p_j,\ j = x, y, z \qquad (13)$$

in the lab system,[9] some of the coincidence counts of Coulomb momentum mapping may arise from $C^+D^+$ fragments of two distant $C_2D_2^{2+}$ dications instead of the same parent cation. These accidental coincidences must be further filtered out from the dataset in order to have a clean interpretation of the Coulomb momentum mapping experiment for the making of a molecular movie. To improve interpretation of the Coulomb momentum mapping technique, we need to introduce an additional criterion to ensure that the coincidence counts originate from fragments of the same parent ion by filtering out the coincidence with relative momentum of two ion particles which are lower than would be allowed by reasonable bond length between these two particles in their parent ion. This

novel criterion basically reflects the essence of Coulomb explosion imaging of bond length.

E. Vibrational coherence produced by sudden change of frequency

After ionization, the vibrational frequencies will change due to the new electronic potential. This is the origin of the oscillation observed in Figure 4b of the main text (with a 27fs period, which is half the vibrational period of symmetric bending), as discussed here. Considering the coherent states that are closely correlated with classical particles in harmonic potential, the vibrational motion can be described by a time-dependent Hamiltonian

$$H = \hbar\omega(t)(a^{+}a + \frac{1}{2}) \ , \tag{14}$$

where

$$\omega(t) = \begin{cases} \omega_0, \ t \le 0 \\ \omega_1, \ t > 0 \end{cases} \ , \tag{15}$$

i.e. the frequency undergoes a sudden change at $t$=0 due to ionization. In the Heisenberg picture, we have

$$\begin{aligned} a(t) &= e^{iHt/h} a e^{-iHt/h} = e^{-i\omega_1 t}\left( \frac{\omega_1 + \omega_0}{2\sqrt{\omega_1\omega_0}} a + \frac{\omega_1 - \omega_0}{2\sqrt{\omega_1\omega_0}} a^{+} \right) = U(t)a + V(t)a^{+} \\ a^{+}(t) &= U^{*}(t)a^{+} + V^{*}(t)a \end{aligned} .$$

And we thus have for the collective vibrational amplitude

$$\begin{aligned} \left\langle Q^2 \right\rangle(t) &= \frac{\hbar}{2M\omega_1}\left\langle (a(t) + a^{+}(t))^2 \right\rangle \\ &= \frac{\hbar}{M\omega_1}\left\langle \left| U(t) + V^{*}(t) \right|^2 (n + \frac{1}{2}) \right\rangle \\ &= \frac{\hbar}{4M\omega_0}\coth\frac{\hbar\omega_0}{2k_B T}\left[ \left(1 + (\omega_0/\omega_1)^2\right) + \left(1 - (\omega_1/\omega_0)^2\right)\cos(2\omega_1 t) \right] \end{aligned} , \tag{16}$$

where $T$ is temperature, $M$ is reduced mass of the vibrational mode, and $k_B$ is the Boltzmann constant.

## Supplementary References


1 Gilbert, A. T. B., Besley, N. A. & Gill, P. M. W. Self-Consistent Field Calculations of Excited States Using the Maximum Overlap Method. *J. Phys. Chem. A* **112**, 13164-13171, (2008).

2 Gadea, F. X., Köppel, H., Schirmer, J., Cederbaum, L. S., Randall, K. J., Bradshaw, A. M., Ma, Y., Sette, F. & Chen, C. T. *Phys. Rev. Lett.* **66**, 883, (1991).

3 Manne, R. & Ågren, H. Auger transition amplitudes from general many-electron wavefunctions. *Chem. Phys.* **93**, 201-208, (1985).

4 Inhester, L., Burmeister, C. F., Groenhof, G. & Grubmüller, H. Auger spectrum of a water molecule after single and double core ionization. *J. Chem. Phys.* **136**, 144304, (2012).

5 Kivimäki, A., Neeb, M., Kempgens, B., Köppe, H. M., Maier, K. & Bradshaw, A. M. Angle-resolved Auger spectra of the C2H2 molecule. *J. Phys. B* **30**, 4279, (1997).

6 Siegbahn, H., Asplund, L. & Kelfve, P. The Auger electron spectrum of water vapour. *Chem. Phys. Lett.* **35**, 330, (1975).

7 Son, S.-K., Young, L. & Santra, R. Impact of hollow-atom formation on coherent x-ray scattering at high intensity. *Phys. Rev. A* **83**, 033402, (2011).

8 Osipov, T., Rescigno, T. N., Weber, T., Miyabe, S., Jahnke, T., Alnaser, A. S., Hertlein, M. P., Jagutzki, O., Schmidt, L. P. H., Schöffler, M., Foucar, L., Schössler, S., Havermeier, T., Odenweller, M., Voss, S., Feinberg, B., Landers, A. L., Prior, M. H., Dörner, R., Cocke, C. L. & Belkacem, A. Fragmentation pathways for selected electronic states of the acetylene dication. *J. Phys. B* **41**, 091001, (2008).

9 Liekhus-Schmaltz, C. E., Tenney, I., Osipov, T., Sanchez-Gonzalez, A., Berrah, N., Boll, R., Bomme, C., Bostedt, C., Bozek, J. D., Carron, S., Coffee, R., Devin, J., Erk, B., Ferguson, K. R., Field, R. W., Foucar, L., Frasinski, L. J., Glownia, J. M., Guhr, M., Kamalov, A., Krzywinski, J., Li, H., Marangos, J. P., Martinez, T. J., McFarland, B. K., Miyabe, S., Murphy, B., Natan, A., Rolles, D., Rudenko, A., Siano, M., Simpson, E. R., Spector, L., Swiggers, M., Walke, D., Wang, S., Weber, T., Bucksbaum, P. H. & Petrovic, V. S. Ultrafast isomerization initiated by X-ray core ionization. *Nature Comm.* **6**, 8199, (2015).

10 Osipov, T., Cocke, C. L., Prior, M. H., Landers, A., Weber, T., Jagutzki, O., Schmidt, L., Schmidt-Böcking, H. & Dörner, R. Photoelectron-Photoion Momentum Spectroscopy as a Clock for Chemical Rearrangements: Isomerization of the Di-Cation of Acetylene to the Vinylidene Configuration. *Phys. Rev. Lett.* **90**, 233002, (2003).

11 Glover, W. J. *J. Chem. Phys.* **141**, 171102, (2014).

12 Ben-Nun, M. & Martínez, T. J. Ab Initio Quantum Molecular Dynamics. *Adv. Chem. Phys.* **121**, 439-512, (2002).

13 Hohenstein, E. G., Luehr, N., I. S. Ufimtsev & Martinez, T. J. An atomic orbital-based formulation of the complete active space self-consistent field method on graphical processing units. *J. Chem. Phys.* **142**, 224103, (2015).

14 Snyder, J. W., Hohenstein, E. G., Luehr, N. & Martinez, T. J. An atomic orbital-based formulation of analytical gradients and nonadiabatic coupling vector elements for the state-averaged complete active-space self-consistent field method on graphical processing units. *J. Chem. Phys.* **143**, 154107, (2015).

15 Zyubina, T. S., Dyakov, Y. A., Lin, S. H., Bandrauk, A. D. & Mebel, A. M. Theoretical study of isomerization and dissociation of acetylene dication in the ground and excited electronic states. *J. Chem. Phys.* **123**, 134320, (2005).

16 Palaudoux, J., Jutier, L. & Hochlaf, M. Theoretical spectroscopy of acetylene dication and its deuterated species. *J. Chem. Phys.* **132**, 194301, (2010).

17 Scharma, A. R., Bowman, J. M. & Nesbitt, D. J. *J. Chem. Phys.* **136**, 034305, (2012).
